# The Comparison of the Efficiency of Emodin and Aloe-Emodin in Photodynamic Therapy

**DOI:** 10.3390/ijms23116276

**Published:** 2022-06-03

**Authors:** Martyna Nowak-Perlak, Mariusz A. Bromke, Piotr Ziółkowski, Marta Woźniak

**Affiliations:** 1 Department of Clinical and Experimental Pathology, Division of General and Experimental Pathology, Wroclaw Medical University, 50-368 Wroclaw, Poland; martyna.nowak-perlak@student.umw.edu.pl (M.N.-P.); piotr.ziolkowski@umw.edu.pl (P.Z.); 2 Department of Biochemistry and Immunochemistry, Wroclaw Medical University, 50-368 Wroclaw, Poland; mariusz.bromke@umw.edu.pl

**Keywords:** skin cancer, photodynamic therapy, natural compound, emodin, aloe-emodin

## Abstract

Skin cancer (melanoma and non-melanoma) is the most frequent type of malignancy in the Caucasian population. Photodynamic therapy (PDT) as an interesting and unique strategy may potentially boost standard therapeutic approaches. In the present study, the potential of emodin and aloe-emodin as photosensitizers in photodynamic therapy has been investigated. The conducted research presents for the first-time comparison of the phototoxic and anti-cancerous effects of emodin and aloe-emodin on skin cancer cell lines, including SCC-25 representing cutaneous squamous cell carcinoma, MUG-Mel2 representing a melanoma cell line, and normal human keratinocytes HaCaT representing control normal skin cells. To assess the effectiveness of emodin and aloe-emodin as a photosensitizer in PDT on different skin cell lines, we performed MTT assay measuring cytotoxicity of natural compounds, cellular uptake, apoptosis with flow cytometry, and a wound-healing assay. Although emodin and aloe-emodin are isomers and differ only in the position of one hydroxyl group, our phototoxicity and apoptosis detection results show that both substances affect skin cancer cells (SSC-25 squamous cell carcinoma and MUG-Mel2 melanoma) and normal keratinocytes (HaCaT cell line) in other ways. In conclusion, our study provides evidence suggesting that emodin and aloe-emodin mediated PDT exhibits the potential for clinical development as a new effective and safe photosensitizer to treat skin cancer.

## 1. Introduction

Skin cancer (melanoma, and non-melanoma) is the most frequent type of malignancy in the Caucasian population [1]. In other populations, according to epidemiologic studies, skin cancer is the most common type of cancer and has a higher frequency and death rate than other diseases. Skin cancer is caused by mutations in cancer-related genes in skin cells, such as proto-oncogenes and tumor suppressors, leading to a change in cell homeostasis and uncontrolled cutaneous cell proliferation [2]. Current therapies for the treatment of skin tumors contain chemotherapy, immunotherapy, radiotherapy, and targeted therapy. The treatments outlined above have a few drawbacks: they are costly, very toxic, and, in some circumstances, unsuccessful because of resistance, particularly in metastatic forms [3]. The patients often are offered surgical excision of skin cancers. In most situations, excision is quite successful. Nevertheless, the choice to conduct surgery might be influenced by a variety of factors, including the patient’s co-morbidities or probable tissue intolerance. Patients with field cancerization or many clinical and sub-clinical lesions across a vast region of the body are frequently prescribed topical treatment. Local therapies may also allow for greater pharmaceutical levels at the tumor site while posing a lower risk of harm for the patient than systemic therapies [4]. Therefore, it is important to discover more efficient therapeutic strategies that are still required, which have fewer side effects for the patients.

Photodynamic therapy (PDT) is an interesting and unique strategy that might potentially overcome the above-described limitations. The primary idea behind this procedure is to apply and accumulate the specified compound with photosensitive properties (photosensitizer—PS) into tumor tissue. Following local irradiation with a certain wavelength laser, activated PS can convert neighboring molecules into highly active reactive oxygen forms (ROS) [5]. We can distinguish two types of reactions. The first mechanism involves the direct transfer of energy from photosensitizer to oxygen molecules dissolved in cells, resulting in the creation of singlet oxygen, which is toxic to cells. The second mechanism involves direct interaction with a cell membrane or another molecule, which results in the transport of an electron to generate radicals that react with oxygen and create oxygen molecules [6].

Oxidative stress is a condition in which cellular components such as DNA, proteins, and lipids are oxidized and harmed as a result of an increase in ROS. The ability of cells to endure stress and remove or repair damaged parts determines the exposed organism’s survival. In response to oxidative assaults, many stress response mechanisms are activated, including the activation of enzymatic and non-enzymatic antioxidizing agents. On the other hand, ROS triggers a variety of intracellular signaling pathways and can result in a variety of outcomes, including cell death and growth inhibition. Photokilling can emerge under harsh conditions of necrosis or in milder forms of apoptosis and autophagy, depending on the efficacy of the photosensitizer. The type of cell death depends on the stimulus, the intensity, and the amount of photosensitizer. The above factors determine the kind of photokilling [7,8].

Due to its low invasiveness, excellent selectivity, and relatively lower costs than other therapies, photodynamic therapy is gaining popularity. Because skin cancers are easily accessible and easy to irradiate, their localization is beneficial in PDT. In comparison to traditional radiotherapy and chemotherapy, PDT offers fairly strong selective anti-tumor activity and low side effects due to a control of the light exposure region and PS activation on-demand. PDT also promotes anti-tumor immunity and the formation of immunological memory, which helps to prevent tumor growth [6,9].

Emodin (1,3,8-trihydroxy-6-methylanthraquinone) is a natural anthraquinone derivative found in a variety of popular Chinese medicinal plants. This substance has been practiced in traditional Chinese medicine for over 2000 years and may still be found in a wide variety of herbal products. It is present in a wide range of Higher Plant taxa, including those from the *Rhamnaceae*, *Polygonaceae*, *Fabaceae*, and *Asteraceae* families. Emodin is also found in the fungus genera *Aspergillus*, *Cladosporium*, *Chaetomium*, *Penicillium*, and *Penicilliosis* as a red color. The chemical can be observed in nature in its free form, as a red-orange powder or crystals, and as glycosides. Its biological role in plants appears to be connected to the compound’s capacity to protect vegetable organs against herbivores, diseases, competition, and a variety of other external abiotic stimuli such as strong sunlight [10,11]. Emodin has a wide variety of pharmacological activities, encompassing anticancer, anti-inflammatory, antioxidant, antibacterial, antiviral, anti-diabetes, immunosuppressive, and osteogenesis promoting effects [12]. Scientists confirm the anticancer effect of emodin among others in neuroblastoma, hepatocellular carcinoma, lung adenocarcinoma, pancreatic cancer, breast cancer, melanoma cells, and squamous cell carcinoma [13,14,15,16,17,18,19].

Aloe-emodin (1,8-dihydroxy-3-(hydroxymethyl)-anthraquinone) is an anthraquinone that is one of several bioactive components found in *Aloe vera*, a perennial cactus-like plant that grows in tropical regions all over the world. It has been used as a traditional Chinese medicine for centuries, and it is still incredibly useful among cancer and non-cancer patients [20]. Aloe-emodin has a variety of therapeutic biochemical activities, including anti-inflammatory, immunoregulation, and wound healing stimulation. Aloe-emodin has been shown to have anti-carcinogenic properties in a range of malignancies by suppressing cell proliferation, migration, and invasion [21]. Anticancer activity of aloe-emodin has been reported in neuroectodermal tumors, lung squamous cell carcinoma, hepatoma cells, colon carcinoma cells, melanoma, and squamous cell carcinoma [21,22,23,24,25,26].

Interestingly, the latest research shows that plant-derived substances emodin and aloe-emodin are also used in COVID-19 [27,28]. Emodin and aloe-emodin due to their unique properties might play a significant role in PDT, acting as both photosensitizers and a direct therapeutic molecule. Experiments conducted in vitro and in vivo suggest that emodin and aloe-emodin can alter a variety of molecular responses in inflammatory pathways, including the production of ROS, the upregulation of caspase-3 and Bax protein levels, the downregulation of Bcl-2 protein levels, and the overexpression of genes involved in cell death mechanisms [29,30]. Therefore, emodin and aloe-emodin may raise the likelihood of apoptosis and necrosis in faulty cells while also stimulating the creation of cell-killing radicals, making plant-derived substances promising compounds for PDT therapy.

**Figure 1:**
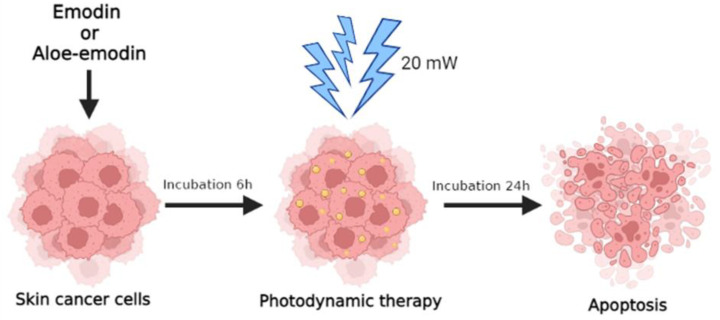
Scheme of emodin or aloe-emodin treated and mechanism of photodynamic therapy.

In this study, we conducted a comparison of the phototoxic and anti-cancerous effects of emodin and aloe-emodin on skin cancer cell lines, including SCC-25 representing cutaneous squamous cell carcinoma, MUG-Mel2 representing a melanoma cell line, and normal human keratinocytes HaCaT representing control normal skin cells. To assess the effectiveness of emodin and aloe-emodin as a photosensitizer in PDT on different skin cell lines, we performed MTT assay measuring cytotoxicity of natural compounds, cellular uptake using mass spectrometry, apoptosis rate with flow cytometry, and wound-healing assay (Figure 1) to validate proliferation and migration capacity after the proposed treatment.

## 2. Results

### 2.1. The Effect of Emodin and Aloe-Emodin on HaCaT, SCC-25, and MUG-Mel2 Cells in MTT Assay

**Figure 2:**
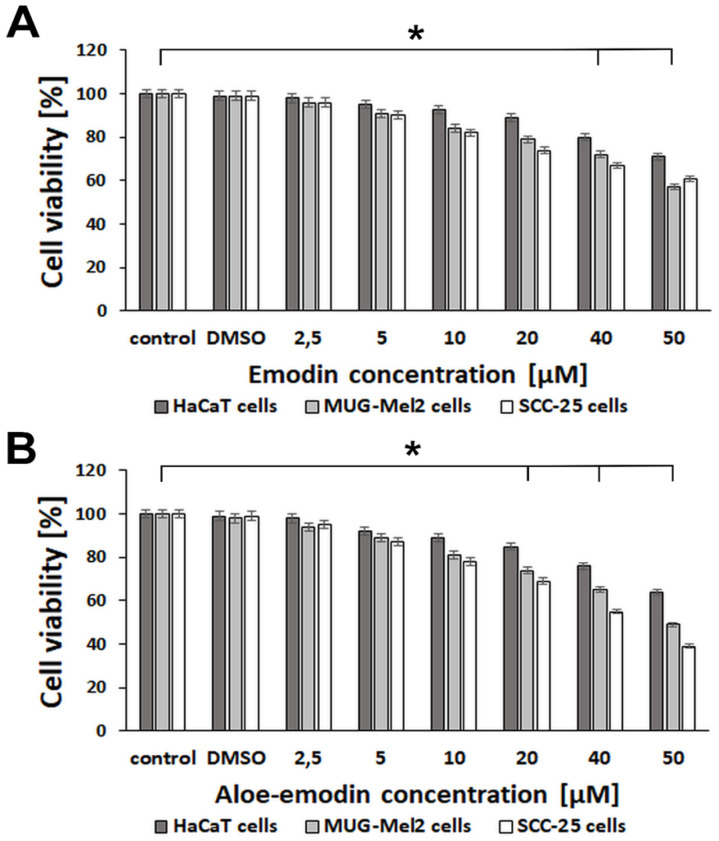
Comparison of HaCaT, MUG-Mel2, and SCC-25 cells viability after 24 h incubation with (**A**) emodin or (**B**) aloe-emodin at concentrations of 2.5–50 μM. Results represent the mean from three different experiments from four wells. * *p* < 0.05.

The effect of emodin and aloe-emodin was performed on normal keratinocyte cells HaCaT, skin cancer cells MUG-Mel2 (melanoma cells), and SCC-25 (squamous cell carcinoma). The effectiveness of emodin (Figure 2A) and aloe-emodin (Figure 2B) was compared in doses of 2.5, 5, 10, 20, 40, and 50 μM. The results showed that aloe-emodin reduced viability more than emodin in both cancer cell types. For instance, emodin in 20 μM concentration caused decreased viability in MUG-Mel2 (79%) and SCC-25 (74%), whereas aloe-emodin caused higher cell death in MUG-Mel2 (74%) and SCC-25 (69%). The viability of HaCaT (normal keratinocytes) was decreased only by 11% in emodin and 15% in aloe-emodin at 20 μM concentration. Interestingly, HaCaT cells maintained higher viability after different treatments than cancer cell lines. In the next experiment, we used 20 μM and 40 μM concentrations of emodin and aloe-emodin with blue light irradiation to examine the influence of these natural substances in photodynamic therapy on cells.

### 2.2. The Effect of Emodin-Based PDT and Aloe-Emodin-Based PDT on HaCaT, SCC-25, and MUG-Mel2 Cells in MTT Assay

**Figure 3:**
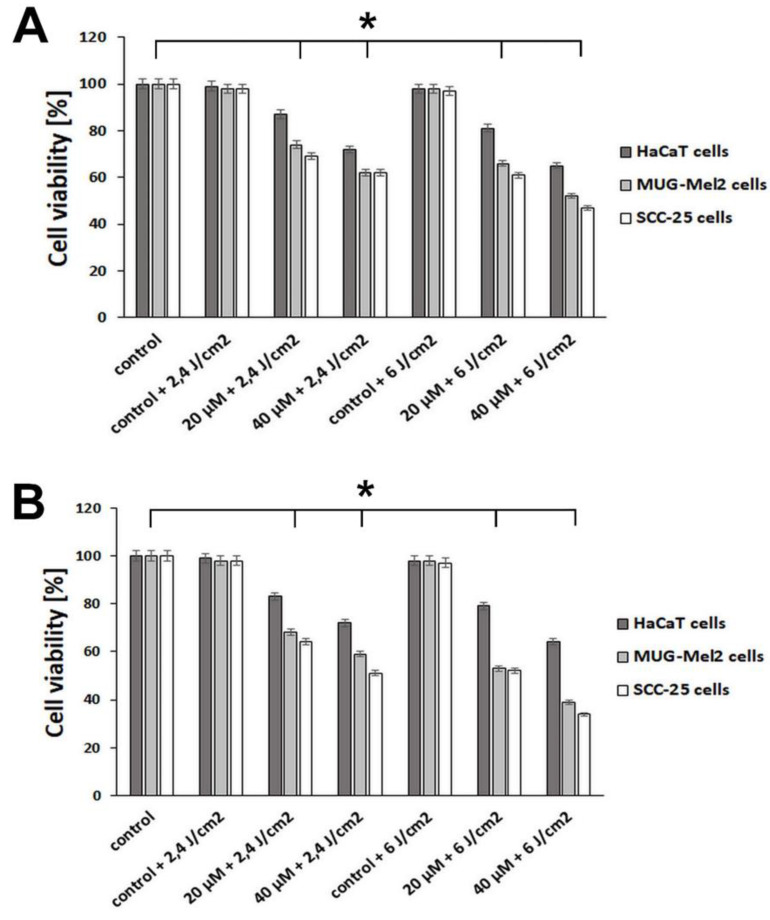
Comparison of HaCaT, MUG-Mel2, and SCC-25 cells viability after 24 h incubation with (**A**) emodin-based PDT or (**B**) aloe-emodin-based PDT at concentrations of 20 μM and 40 μM after blue light low irradiation (2.4 J/cm^2^) or medium irradiation (6 J/cm^2^). Results represent the mean from three different experiments from four wells. * *p* < 0.05.

The effect of emodin-based PDT and aloe-emodin-based PDT was performed on normal keratinocyte cells HaCaT, skin cancer cells MUG-Mel2 (melanoma cells), and SCC-25 (squamous cell carcinoma). The effectiveness of these two natural substances with irradiation was compared in doses of 20 and 40 μM after blue light low irradiation (2.4 J/cm^2^) or medium irradiation (6 J/cm^2^). We can observe that natural substances (emodin and aloe-emodin) and irradiation cause a higher reduction of viability in cell lines than alone natural substances without irradiation. Results indicated that aloe-emodin-based PDT (Figure 3B) caused a higher reduction of cell viability in both cancer cell lines than emodin-based PDT (Figure 3A). For instance, emodin in 20 μM concentration and low irradiation caused decreased viability in MUG-Mel2 (74%) and SCC-25 (69%), but emodin in the same concentration and high irradiation caused higher cell death in MUG-Mel2 (66%) and SCC-25 (61%). In comparison, aloe-emodin in 20 μM concentration and low irradiation caused decreased viability in MUG-Mel2 (68%) and SCC-25 (64%), and aloe-emodin in the same concentration and irradiation caused around 10% higher cell death in MUG-Mel2 (53%) and SCC-25 (52%). The viability of HaCaT (normal keratinocytes) was decreased only by 13% in emodin (20 μM) and low irradiation and 19% in average irradiation. Using aloe-emodin (20 μM) and low irradiation, cell viability decreased by 17% and aloe-emodin and average irradiation by 21%. Emodin and aloe-emodin in the concentration of 20 μM and medium irradiation (6 J/cm^2^) were chosen in all subsequent biological studies.

### 2.3. The Effect of Emodin-Based PDT and Aloe-Emodin-Based PDT on HaCaT, SCC-25, and MUG-Mel2 Cells in the Wound-Healing Process

**Figure 4:**
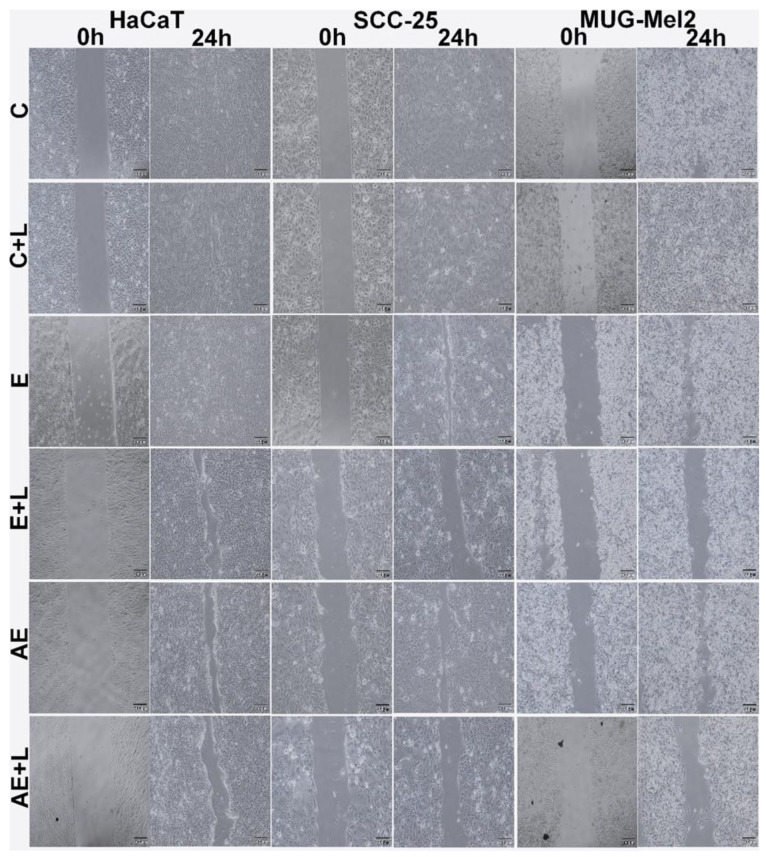
Wound-healing assay in time point 0 h and 24 h of HaCaT, SCC-25, and MUG-Mel2 cell line. Representative images show that, in SCC-25 and MUG-Mel2 after 24 h, the scrap in control cells is minimal compared to treated cells with emodin (E) or aloe-emodin (AE) in dose 20 µM with blue light (L) (6 J/cm^2^). In treated cells, in the HaCaT control cell line, the scrap is smaller than in the other two cancer cells. Scale bar = 200 µm.

The wound healing test was used to check whether emodin-based PDT and aloe-emodin-based PDT decrease the motility of HaCaT, SCC-25, and MUG-Mel2 cells. The experiment demonstrates cell migration by measuring the closure of a primordial scratch after 24 h. If the motility of cells is small (compound causes cytotoxic effect and cell death), we can observe a wide gap (wound) between cells. On the other hand, if the motility of cells is not compromised (no cytotoxicity, cells still alive), we can observe the overgrown, narrow gap between cells. The results demonstrate that PDT with aloe-emodin caused the strongest effect of migration properties in MUG-Mel2 cancer cells. After 24 h of the treatment, there was no migration observed in MUG-Mel2. On the other hand, minimal migration after emodin-based PDT was observed in MUG-Mel2. In SCC-25 cells treated with emodin-based PDT, the wound was minimally closed, whereas, in SCC-25 treated with aloe-emodin-based PDT, minimal migration was seen In normal HaCaT cells, the wound closed almost entirely within 24 h of incubation after therapy. The results are presented in Figure 4.

### 2.4. Tunel Assay—The Impact of Emodin, Emodin-Based PDT, Aloe-Emodin, and Aloe-Emodin-Based PDT on SCC-25 and MUG-Mel2 Cell Lines

**Figure 5:**
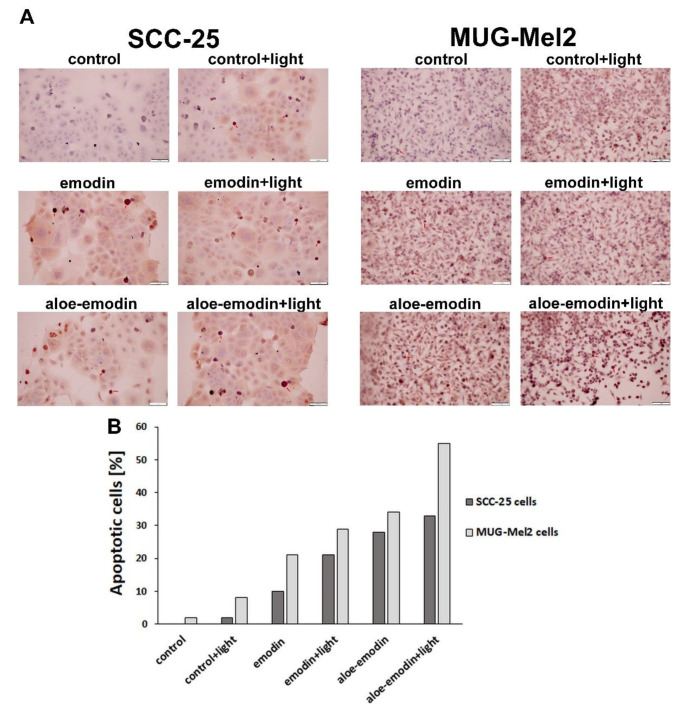
Results from TUNEL assay for SCC-25, and MUG-Mel2 cell line followed photodynamic therapy (PDT) with emodin or aloe-emodin at a dose of 20 µM with the blue light (6 J/cm^2^) (**A**). Diagram shows the rate of TUNEL-positive cells (peroxidase-positive) determined by dividing the number of TUNEL-positive cells by the total number of cells in the slides × 100% (**B**). Scale bar = 100 µm.

The TUNEL assay was used to detect apoptosis in SCC-25 and MUG-Mel2 cells after treatment with emodin-followdPDT and aloe-emodin-followed PDT. The TUNEL method is based on the ability of terminal deoxynucleotidyltransferase (TdT) to label blunt ends of double-stranded DNA breaks independent of a template. The brown color of peroxidase indicates TUNEL-positive apoptotic cell death through condensation of chromatin and cell blebbing. We have marked apoptotic cells with red arrows in Figure 5A. Figure 5 presents peroxidase an in situ TUNEL method, hematoxylin-counterstained. The results demonstrate that PDT with aloe-emodin caused the strongest apoptotic effect in MUG-Mel2 cancer cells (60% of apoptotic cells). In SCC-25 cells treated with emodin-based PDT, 20% of apoptotic cells were detected. Whereas, treated with aloe-emodin-based PDT, it was more than 30% of apoptotic cells.

### 2.5. Apoptosis Assay—The Impact of Emodin, Emodin-Based PDT, Aloe-Emodin, and Aloe-Emodin-Based PDT on SCC-25 and MUG-Mel2 Cell Lines

**Figure 6:**
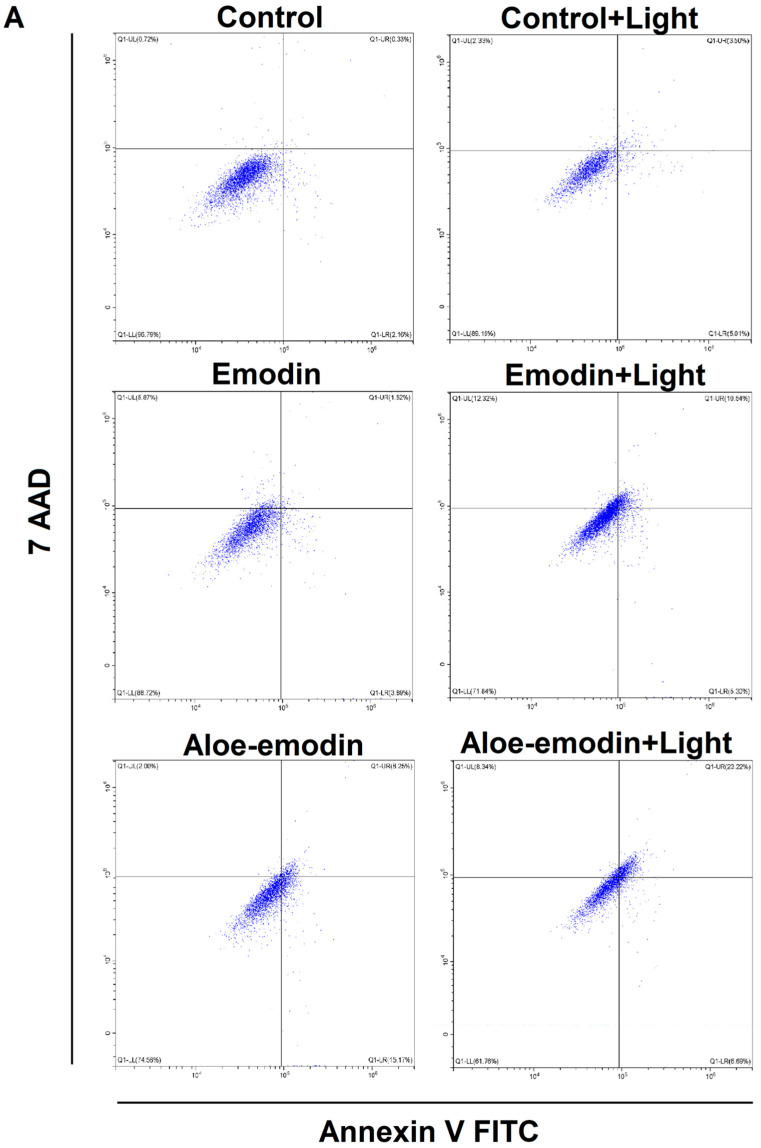

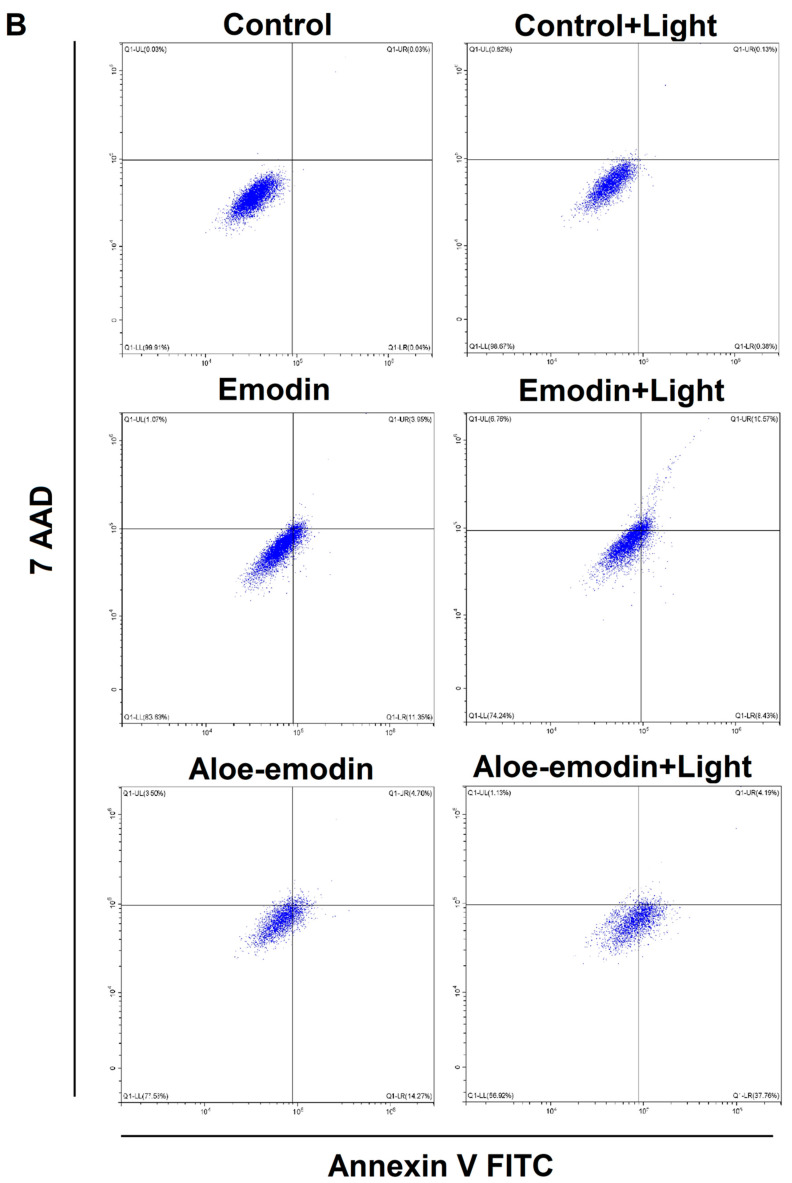
Effect of photodynamic therapy (PDT) with emodin or aloe-emodin in dose 20 µM with the blue light (6 J/cm^2^) in SCC-25 (**A**); MUG-Mel2 (**B**) cells. After treatment, the cells were stained using Annexin-FITC/7-AAD Kit and were measured by flow cytometry.

Flow cytometry analysis evaluated cell death caused by emodin or aloe-emodin and emodin-based PDT or aloe-emodin-based PDT in SCC-25 and MUG-Mel2. Early and late apoptosis and necrosis were observed in SCC-25 and MUG-Mel2 cells after 24 h of treatment, as demonstrated in Figure 6. Minimal cell death was shown in control cells. In SCC-25, the compound of emodin or aloe-emodin promoted cell death by around 8% and 22%, accordingly. In MUG-Mel2 cells, emodin or aloe-emodin increased cell death respectively by about 16% and 22% in comparison to control cells. Interestingly, in SCC-25, the combination emodin-based PDT or aloe-emodin-based PDT showed cell death, respectively 25% and 35% as compared to control cells. The combination of emodin-based PDT or aloe-emodin-based PDT boosted cell death by around 26% and 43% in MUG-Mel2, respectively. Notably, after 24 h from irradiation, in MUG-Mel2, cell death is mainly caused by early and late apoptosis, whereas, in SCC-25, cell death is primarily due to late apoptosis and necrosis.

### 2.6. Results of Cellular Uptake with Emodin and Aloe-Emodin on SCC-25 and MUG-Mel2 Cell Lines

The uptake of emodin and aloe-emodin was studied with the use of liquid chromatography-mass spectrometry (LCMS). Both compounds have the same molecular mass and were detected as ions 269.045 m/z but differed significantly in their retention time. Elution time for the standard of aloe-emodin was 5.05 min., whereas emodin eluted at 7.8 min. For verification, specific daughter ions in MSE mode (similar to MSMS) were followed as well, 240.04 m/z and 225.03 m/z, respectively.

**Figure 7:**
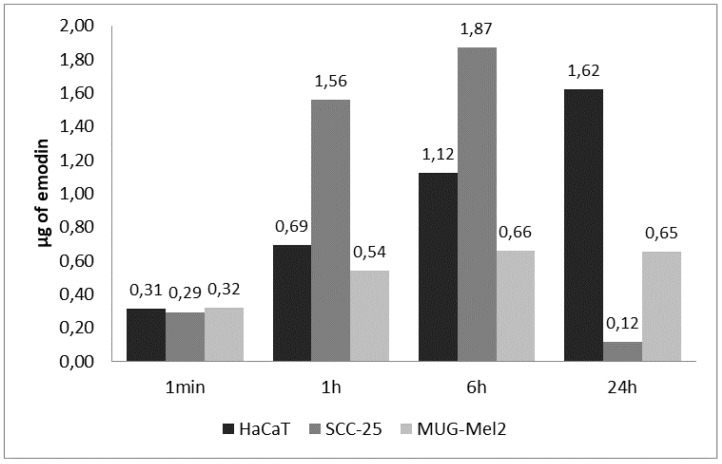
Emodin extracted from different cell lines treated with 30 µM emodin in growth medium.

Extracts of treated cells were prepared by lysing cells with 1 mL of methanol. To ensure that the extracts contained emodin (or aloe-emodin), which was actually taken up by the cells, after the removal of growth media and prior to the extraction, cells were washed twice with PBS. Samples of medium, PBS from the second wash, and extracts were analyzed. On average, concentration of emodin in washing, PBS represented 3.3 % of the concentration of the emodin in the corresponding extract. This result suggests that the emodin found in extracts was largely taken-up by the treated cells. The content of emodin found in three cell lines over the time of 24 h is presented in Figure 7. Already after one hour, SCC-25 accumulated significant amounts of emodin. Interestingly, these cells contained very little amounts of emodin after 24 h. In parallel, we have observed significant reductions of free emodin in the medium for SCC-25 after 24 h (Supplementary Data). Moreover, in these samples, we have observed the appearance of chromatographic peaks for ions 269.045 m/z but with a smaller retention time. These ions co-eluted with 445.07 m/z at 4.9 min. Characteristic mass difference (176.03 m/z) might suggest active, especially in SCC-25, biotransformation (glucuronidation) of emodin. HaCaT cells displayed a steady increase in the content of emodin, whereas emodin extracted from MUG-Mel2 did not differ much during the experiment time points.

**Figure 8:**
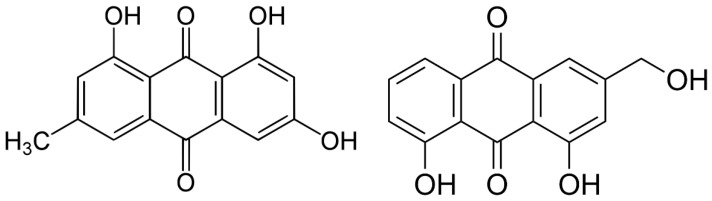
The structure of Emodin (**on the left**) and Aloe-emodin (**on the right**).

To compare uptake of emodin and aloe-emodin (Figure 8), studied cells were grown in parallel inappropriate media supplemented with 30 µM of either substance. The incubation was carried over 6 h, after which cells were washed and extracted (Table 1). No significant difference between in content of emodin and aloe-emodin is observed. Only from MUG-Mel2 cells could consistently higher amounts of aloe-emodin than emodin be extracted in both replicates, but the difference is not significant. Nevertheless, a 30 µM (approximately 8.1 µg/mL) concentration of emodin and aloe-emodin is sufficient to induce growth inhibition in various cell types.

## 3. Discussion

In previous years, many experts from the photodynamic therapy field have focused on the different plant extracts or natural compounds as a new strategy for discovering effective photosensitizers [31]. Numerous light-sensitive substances were examined for their efficient phototoxic action toward neoplastic cells and less cytotoxic action on normal cells. Several research studies focused on curcumin, a natural phytochemical derived from the rhizome of turmeric (*Curcuma longa* L.) [5,32]. However, due to its poor cellular availability, rapid metabolism, and other pharmacokinetic characteristics, its use in cancer treatment is limited. Therefore, new natural photosensitizers with confirmed stability, improved bioavailability for cancer cells, but less toxic to normal cells, and, most importantly, extremely effective in photokilling of malignant cells are still needed.

In the present study, the potential of emodin and aloe-emodin as photosensitizers in photodynamic therapy has been investigated on three skin cell lines: squamous cell carcinoma SCC-25, melanoma MUG-Mel2, and immortalized keratinocytes HaCaT cells. According to Yunqing Liu et al. and Galiardi-Campoy et al., both natural anthraquinones show beneficial phototoxic properties against human oral mucosa carcinoma and cervical carcinoma cells, respectively [29,30]. Considering the promising role of the above-described agents in PDT, the aim of the study was to compare the effectiveness of both substances in photokilling of skin cancer cells after low and high doses of the blue light irradiation, to measure their ability to accumulate in three cell lines, to generate apoptosis and impact cells’ capacity to proliferate and migrate after proposed therapy.

According to Liu et al. [29] and Zang et al. [33], emodin and aloe-emodin mediated PDT, respectively, both have absorption peaks between 430–480 nm. Therefore, for this study, authors decided to use blue light to irradiate cells. Moreover, Vargas et al. [34] investigated and confirmed that emodin and aloe-emodin are phototoxic to red blood cells used in their experiments. The analyzed substances after irradiation involved radical species and reactive oxygen species in the phototoxic mechanism.

The most common wavelengths used in the clinical treatment are 630 nm (red light PDT) or 450 nm (blue light PDT). Light sources of shorter wavelengths are proposed for the treatment of superficial diseases. Nevertheless, a pilot study of BCC patients conducted by Maytin et al. [35] reveals that blue light and red light PDT appear to be equally safe for the treatment of BCC skin cancer. Therefore, blue light active photosensitizers may also be a potential treatment option.

To the best of the authors’ knowledge, emodin, and aloe-emodin-mediated PDT were compared on skin cancer cells and normal keratinocytes for the first time. Our initial results indicated that both substances reveal lower cytotoxicity towards normal keratinocytes in comparison to skin cancer cell lines. The MTT experiments were conducted in a broad range of doses of the natural substance—from 2.5 µM to 50 µM for 24 h. According to the findings of Colombo et al. [36], these immortalized adult human keratinocytes have been proposed as a model cell line for studying normal keratinocyte activities because HaCaT cells maintained in a culture medium without calcium show normal morphogenesis, and the expression of cellular membrane markers [37].

The statistically significant difference in keratinocyte viability (around 80%) was observed in a concentration of 20 µM for both analyzed agents after irradiation (Figure 2 and Figure 3), whereas, in malignant cell lines, the viability was decreased by almost 50%. Interestingly, Pecere et al. [22] observed that, in mice with severe combined immunodeficiency, the development of human neuroectodermal tumors is suppressed by aloe-emodin without causing significant toxicity. The authors demonstrated that this natural substance does not have an inhibitory effect on normal fibroblasts or hemopoietic progenitor cells. The presented observations also correspond to our previous study focused on the efficacy of curcumin-blue light-mediated PDT on skin cancer cells and normal keratinocytes. The proposed therapy revealed lower phototoxicity against normal skin cells in comparison to malignant cells [5].

To explore the mechanism by which emodin and aloe-emodin lead to cell death, flow cytometry and TUNEL assay were performed to examine the rate of early, late apoptotic, and necrotic cells. Both cancer cells demonstrate that PDT with aloe-emodin caused a stronger apoptotic effect than emodin. Furthermore, melanoma cells reveal twice as many cells damaged by programmed cell death in comparison to squamous cell carcinoma cell lines (60% and 30%, respectively). Moreover, based on a literature review and flow cytometry analysis, it may be clearly observed that aloe-emodin followed irradiation of skin cancer cells boosted cell apoptosis by more than 20% [21,26]. Additionally, as shown in the wound healing experiments, migration and invasion of skin cancer cells decreased more obviously in the PDT-treated groups (Figure 4).

Based on gleaned, MTT, flow cytometry, and wound healing experiments’ results, conclusions emerge that aloe-emodin is a more potent photosensitizer for the photodynamic treatment of melanoma cells. These findings suggested that the cellular uptake analysis may reveal the reason.

Mass spectrometry analysis showed that emodin is being taken up by studied cells. The analysis of emodin in washing liquid suggests very low amounts of the phytochemical being stuck to surfaces, including surfaces of cells. The studied cells utilize different strategies with respect to dealing with emodin available in the growth medium. The main conclusion of the experiment is the confirmation of uptake of emodin and aloe-emodin into cultured cells (Figure 7). Immortalized keratinocytes—HaCaT cells, which displayed the smallest growth inhibition in contact with emodin—accumulated emodin through the time course of the experiment, steadily increasing cellular concentration. On the other hand, MUG-Mel2 seems to utilize a different strategy in which only small amounts of emodin were taken up by the cells. The content of emodin changed little over this period in the case of MUG-Mel2 cells—amounts recovered from extracts after one, six, and 24 h were similar. This is in contrast with emodin-induced growth inhibition of MUG-Mel2 in wound-healing experiments (Figure 5) or apoptosis assays (Figure 6). Interestingly, in our experiments with SCC-25 cells, which were treated with emodin, the squamous carcinoma cells showed minimal signals of apoptosis (Figure 6). This observation goes along with a measured reduction in the content of emodin as well as the appearance of putative glucuronides of emodin in the cell medium. This suggests active phase II biotransformation and removal of the emodin in the form of glucuronides from SCC-25 cells. No corresponding peak of aloe-emodin glucuronide or aloe-emodin sulfate (349.0 m/z) could be detected in the medium of SCC-25. Chen et al. [38] reported that UDP-glucuronosyltransferase 2B7 (UGT2B7) plays a significant role in the biotransformation of emodin by hepatocyte and nephrocyte microsomes. The main form of glucuronide of emodine seems to be emodin-3-O-β-d-glucuronide [39]. The members of the UGT2B enzyme family show overlapping specificities between isoforms, and the relative activities of UGT2B isoforms on emodin and aloe-emodin; their expression in cell lines was not explored in full by now. In MUG-Mel2 and HaCaT cells, we have not detected mass spectrometry signatures of biotransformation products (glucuronides or sulfates) of emodin and aloe-emodin. Drug detoxification in cancer skin cells receives little attention. Only a few reports have been published, including research on UGT expression in melanocytes derived from newborn foreskin tissues, with UGT2B isoforms (UGT2B7, UGT2B10, and UGT2B15) being expressed primarily in normal melanocytes as well as in a primary melanoma cell line [40,41]. This suggests that MUG-Mel2 and HaCaT cells could be expressing phase II biotransformation enzymes. Nevertheless, their activity is on a significantly lower level in comparison to SCC-25 treated with the same concentrations of emodin and aloe-emodin.

As stated above, the inconclusive results for the differences between uptake of emodin and aloe-emodin are a limitation of the study. More experiments with different time points and concentrations should be conducted. Moreover, elucidation of the mechanism of uptake and removal of emodin and aloe-emodin in keratinocytes and melanoma cells will be very important for the development of successful anti-cancer PDT based on these natural anthraquinones.

## 4. Materials and Methods

### 4.1. Cell Culture

HaCaT human epidermal keratinocytes (CLS, Eppelheim, Germany) were cultured in DMEM (Dulbecco’s Modified Eagle Medium) without calcium to maintain normal morphogenesis and expression of the cellular membrane markers, SCC-25-tongue squamous carcinoma (DSMZ, Braunschweig, Germany) cells in DMEM-F12, and Melanoma MUG-Mel2 (DSMZ, Braunschweig, Germany) cells were cultured in RPMI 1640 cell culture medium. The cell culture media were supplemented with 10% fetal bovine serum (FBS), 1% glutamine, and 1% antibiotics. Culture reagents were bought from Gibco (Thermo Fisher Scientific Inc., Waltham, MA, USA). Cells were cultured as a monolayer at the polystyrene 75 mL cell culture flasks (Biologix Cell Culture Flasks, BIOLOGIX EUROPE GmbH, Niederzier, Germany). Cells were maintained at 37 °C and 5% CO_2_ in a humidified atmosphere. The cells were washed in PBS and trypsinized before being used in the experiments (0.025% trypsin and 0.02% EDTA, Sigma-Aldrich, Burlington, MA, USA). For experiments, cells from the 3rd to the 13th passages were used.

### 4.2. Emodin Solution Preparation

The emodin: 6-methyl-1,3,8-trihydroxyanthraquinone (MedChemExpress, Deer Park, Monmouth Junction, NJ, USA) was dissolved in dimethyl sulfoxide (DMSO, Sigma-Aldrich) to prepare 100 mM stock of the substances. Afterwards, the appropriate amount of stock was combined with cell culture medium to reach the needed concentration of the emodin. The concentration of DMSO in the final solutions used for incubation did not exceed 0.05%, and it was confirmed that the maximum concentration had no statistically significant effect on the cells.

### 4.3. Aloe-Emodin Solution Preparation

The aloe-emodin: 1,8-dihydroxy-3-(hydroxymethyl)anthraquinone (MedChemExpress, Deer Park, Monmouth Junction, NJ, USA) was dissolved in dimethyl sulfoxide (DMSO, Sigma-Aldrich) to prepare 100 mM stock of the substances. Afterward, the appropriate amount of stock was combined with a cell culture medium to reach the needed concentration of the aloe-emodin. The concentration of DMSO in the final solutions used for incubation did not exceed 0.05%, and it was confirmed that the maximum concentration had no statistically significant effect on the cells.

### 4.4. PDT Experiment

Cells were incubated with emodin or aloe-emodin in the needed concentration for 6 h according to Chen et al. [42,43] and Li et al. [44]. Then, the wells were washed 2 times with DPBS, fresh medium was added, and irradiation was performed using a halogen lamp (Penta Lamps, Teclas, Lugano, Switzerland) with the radiation power consistency set to 20 mW/cm^2^. The cells were irradiated for 5 min (6 J/cm^2^). The blue light (380−500 nm) was chosen to achieve the photodynamic effect (maximum absorbance emodin at the wavelength of 442 nm [43] and aloe-emodin of 429.3 nm [29]. Cells involved in emodin or aloe-emodin and PDT treatment were protected from light at all times. Cells were maintained in the complete medium during and post-treatment before further assays. After 24 h of irradiation, experiments were conducted according to the protocols.

### 4.5. Cell Viability Assay—MTT Assay

An MTT assay was used to determine the effect of emodin-induced PDT and aloe emodin-induced PDT on cell viability. The MUG-Mel2, SCC, and HaCaT cells (15 × 10^4^ cells/well) grown in a 96-well plate were cultured for 24 h and then experiments were conducted. The cells were cultured as mentioned in the experiment description with emodin or aloe-emodin for 6 h in the dark. Different doses of emodin and aloe-emodin were experimentally established for the next experiments on MUG-Mel2, SCC-25, and HaCaT to obtain IC50. The MTT assay was performed after 24 h of irradiation. The MTT solution was added to the wells at a final concentration of 1 mg/mL for 2 h. Following this, formazan dye was solubilized with 50 μL DMSO and incubation in a shaker at room temperature for 10 min. Absorbance was measured at 490 nm in a BioTek Well-plate Reader (Winooski, VT, USA). The control group absorbance was 100%, whereas the treated samples’ cell viability was counted using the formula: % = (A of experimental wells/A of the control wells) × 100%. After preliminary studies with different emodin and aloe-emodin doses (2.5, 5, 10, 20, and 40 μM) for the MTT assay, emodin and aloe-emodin were chosen in doses of 20 and 40 μM.

### 4.6. Wound Healing Assay

Wound healing assay was performed with the Culture-Insert 2 Well in μ-Dish 35 mm (Ibidi, Grafelfing, Germany). This is an experiment to investigate cell interactions and migration. According to the manufacturer’s instructions, the cells were seeded to obtain the monolayer in both parts of the insert. Following incubation with emodin or aloe-emodin and irradiation, the inserts were removed, the culture medium was exchanged, and the cells were cultured until about 100% confluency was reached in control cells. The control samples were not given any treatment. The images were obtained using a light microscope with a 4x magnifying objective after the inserts were removed at 0 h and after 24 h of incubation (Olympus IX73 with a camera and CellSens Programme, Hamburg, Germany).

### 4.7. Tunel Assay—Apoptosis Assay

In four-well chamber slides (Thermo Fisher Scientific Inc.), cells were plated at a density of 4 × 10^4^ cells per well. Following treatment, the cells were tested in accordance with the manufacturer’s instructions ApopTag Peroxidase in Situ Apoptosis Detection Kit (Merck Millipore, Darmstadt, Germany). In a nutshell, cells were fixed in 1% paraformaldehyde in PBS 7.4 pH for 24 h at 4 °C. After being washed twice with PBS, cells were treated with TdT enzyme for 1 h at 37 °C. The slides were washed three times in PBS before being incubated with anti-digoxigenin peroxidase conjugate for 30 min at room temperature (RT) in a humidified environment, followed by three rinses with PBS at RT. The slides were then treated with the peroxidase substrate, counterstained with hematoxylin, dried, and mounted in a medium. The images were obtained using a light microscope with a 20× magnifying objective (Olympus BX43 with a camera and CellSens Programme, Hamburg, Germany).

### 4.8. Flow Cytometry—Apoptosis Assay

Cells were collected from each well and placed in Eppendorf tubes. Subsequently, cells were centrifuged with PBS washing (5 min, 4 °C, 1000× *g*). The supernatant was blandly removed, and 800 mL of the Binding Buffer were added for every 1 × 10^6^ cell. Following that, 4 μL AAD-7 and 2 μL FITC were added to each sample per the manufacturer’s instructions. Eppendorf tubes were vortexed and incubated at room temperature for 15 min without light. Following incubation, samples were examined on a flow cytometer with the FICT channel for Annexin 5 and the PC5.5 channel for AAD-7 (Cytoflex, Beckman Coulter Life Sciences, Indianapolis, IN, USA). To compensate, negative samples were generated without staining and samples stained with one fluorochrome were created.

### 4.9. Cellular Uptake 

The MUG-Mel2, SCC, and HaCaT cells (1 × 10^6^ cells/well) grown in a 6-well plate were cultured for 24 h prior to the experiment. The appropriate growth medium was supplemented with emodin or aloe-emodin to a final concentration of 30 µM. Cellular accumulation of emodin and aloe-emodin was tested over different times: 1 min., 1 h, 6 h, and 24 h. After the treatment growth, medium was removed and an aliquot was retained as a sample for the uptake analysis. Next, cells were washed twice with 1 mL of PBS. An aliquot from the second wash was taken for analysis. Finally, cells were extracted with 1 mL of methanol. The cells were kept covered with methanol for 30 s. Then, methanol was transferred into an Eppendorf tube and centrifuged for 15 min at 12,000 rpm at 4 °C. Next, the methanolic extract was collected into a new Eppendorf tube for the content analysis. Samples were frozen at −80 °C. Shortly prior to the analysis, samples were thawed, centrifuged, and supernatants without further treatment were transferred into chromatography vials. 

The analysis of the content of emodin and aloe-emodin in treated cells was performed with the use of Acquity UPLC system coupled with Xevo G2 XS (Waters) QToF mass spectrometer. The chromatographic separation was carried on ACQUITY UPLC BEH ShieldRP18, 1.7 µm column. The eluents were water with 0.1% formic acid (A) and methanol with 0.1% formic acid and the flow was 300 µL/min. Then, a 10 min-long gradient was applied: initial 50% B, 7.0 min; 95% B, 9.0 min; 95% B, 9.1 min; 50% B, 10 min; 50% B. Samples were kept at 5 °C in an autosampler and the injection volume was 1 µL. The ESI source was set to negative mode with capillary voltage 3.75 kV, source temperature 150 °C, and desolvation temperature 400 °C. The mass analyzer was set on MSE mode in which a high energy ramp (15.0 to 30.0) generated daughter ions. Emodin, as well as aloe-emodin, were followed as ion 269.045 m/z, with daughter ions 225.03 m/z and 240.04 m/z, respectively. MassLynx software was used to analyze the chromatograms and spectra.

### 4.10. Statistical Analysis

Analysis between the groups was conducted using the ANOVA test for abnormally distributed data, and a *p*-value below 0.05 was considered significant. The PAST program, version 4.03 was used for calculations.

## 5. Conclusions

Although emodin and aloe-emodin are isomers and differ only in the position of one hydroxyl group, our phototoxicity and apoptosis detection results show that both substances affect skin cancer cells (SSC-25 squamous cell carcinoma and MUG-Mel2 melanoma) and normal keratinocytes (HaCaT cell line) in different ways. As judged by its retention time in chromatography, aloe-emodin seems to be less hydrophobic and is a more potent growth inhibitor than emodin after irradiation by blue light in 6 J/cm^2^ dose. Moreover, our results show no significant difference in uptake of these compounds, thus sheer cellular concentration is not sufficient to explain observed significant differences in growth-inhibiting or apoptotic death-inducing potential during PDT. To sum up, more studies are necessary to confirm the potential anticancer activity of aloe-emodin-PDT and shed light on various cellular and molecular mechanisms which are behind our findings.

## Figures and Tables

**Table 1:** Content of emodin and aloe-emodin in three cell lines studied treated with 30 µM of either compound for 6 h. Values represent the mean of two biological replicates.

Cell Line	Emodin [ng]	Aloe-Emodin [ng]
HaCaT	0.90	1.59
SCC-25	1.14	1.32
MUG-Mel2	0.75	1.98

## Supplementary Materials

The following supporting information can be downloaded at: https://www.mdpi.com/article/10.3390/ijms23116276/s1.
